# Causes and characteristics of death in icu: a national study

**DOI:** 10.1186/2197-425X-3-S1-A770

**Published:** 2015-10-01

**Authors:** JC Orban, Y Walrave, M Leone, B Allaouchiche, JY Lefrant, JM Constantin, S Jaber, C Ichai, AzuRea study group

**Affiliations:** Nice University Hospital, Medical Surgical ICU, Nice, France; Anesthesia and Intensive Care Department, APHM Hôpital Nord, Marseille, France; Anesthesia and Intensive Care Department, Hospices Civils de Lyon, Lyon, France; Anesthesia and Intensive Care Department, Nimes University Hospital, Nîmes, France; Anesthesia and Intensive Care Department, Clermont-Ferrand University Hospital, Clermont-Ferrand, France; Anesthesia and Intensive Care Department, Montpellier University Hospital, Montpellier, France

## Introduction

Mortality of ICU patients is a global parameter reported as an end-point in numerous studies. However, causes and characteristics of patients' death are studied only in particular pathologies such as sepsis, cardiac arrest or ARDS ([1, 2]).

## Objectives

The aim of our study was to analyse causes and circumstances of death in a general ICU population.

## Methods

We performed a prospective observational study. Every ICU included all death occurring during a month randomised in the year of the study. Demographic data were collected as well as circumstances of death (organ failure and organ support at this time). An organ failure was defined by a SOFA sub-score ≥ 3. Population of the study was dichotomised in expected death (following withholding or withdrawal of care, or brain death) and unexpected death (following maximal intensity of care). Data are expressed as median and IQR. Comparisons were made by a Mann-Whitney or chi-squared tests as appropriate. A p value < 0.05 was considered as statistically significant.

## Results

Ninety-six ICUs included 698 dead patients during the study time. Main characteristics of the population and their comparison between expected (n=473) and unexpected deaths (n=225) are reported in the table.

At the time of death, 586 (84%) patients presented at least one organ failure: cardiovascular (58%), respiratory (31%), renal (33%), neurologic (30%), liver (8%) and coagulation (8%). At the same time, an organ support was used in 440 (63%) patients: catecholamines (63%), mechanical ventilation (85%), renal replacement therapy (28%) and liver dialysis (1%). Comparison of these parameters between groups is reported in the figure.

## Conclusions

Patients who died in ICU presented, most of the time, at least one organ failure. Expected death patients exhibited more neurologic and respiratory failures whereas cardiovascular failure was more prominent in unexpected death. In the latter group of patients, the proportion of organ support was higher corresponding to a greater intensity of care.

**Figure 1:**
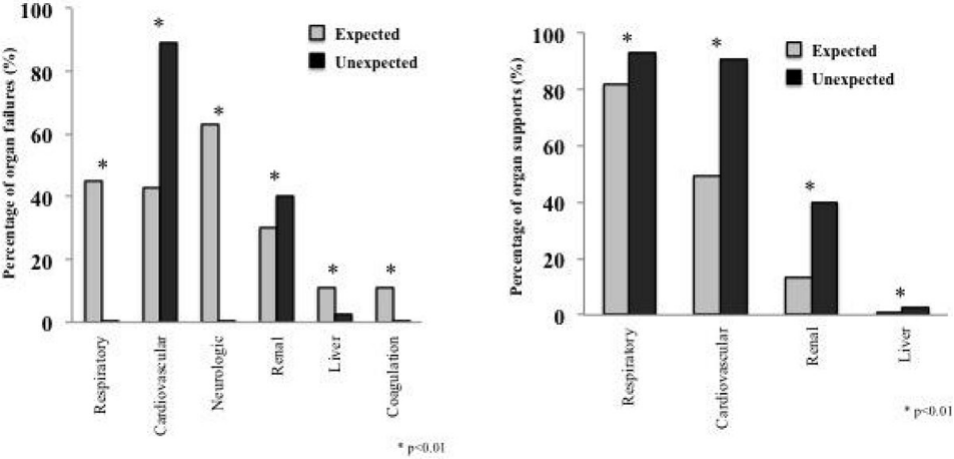
**Percentages of organ failures and support.**

**Table 1:** Demographic data of the population.

	Population of the study	Expected death (n=473)	Unexpected death (n=225)	p values
Age (years)	69 [57-78]	69 [57-78]	68 [56-77]	0.32
SAPS II	63 [48-83]	60 [47-76]	77 [52-93]	< 0.001
SOFA score	11 [7-13]	10 [7-13]	12 [9-15]	< 0.001
Admission causes				
Cardiovascular	193 (28%)	103 (22%)	90 (40%)	< 0.001
Respiratory	182 (26%)	127 (27%)	55 (24%)	0.50
Neurologic	169 (24%)	149 (32%)	20 (9%)	< 0.001
Miscellaneous	154 (22%)	94 (19%)	60 (27%)	0.28
Length of stay (d)	3 [1-10]	5 [2-12]	1 [0-8]	< 0.001
